# Patient with recurrent depressive disorder, Vitamin B12 and folate deficiency after gastric bypass surgery: A Case report

**DOI:** 10.1192/j.eurpsy.2023.1764

**Published:** 2023-07-19

**Authors:** I. Everte, L. H. Konošonoka

**Affiliations:** Strenci Psychoneurological hospital, Strenci, Latvia

## Abstract

**Introduction:**

It is known that after gastric bypass surgery, Vitamin B12 and folate (B9) are common micronutrient deficiencies affecting this population. It may cause several neuropsychiatric symptoms which, if left untreated, leads to severe consequences.

**Objectives:**

To describe a clinical case of the patient with recurrent depressive disorder (RDD), Vitamin B12 and B9 deficiency after gastric bypass surgery, and to review the literature.

**Methods:**

Clinical case presentation through the review of the patient’s clinical file and non-systematic literature review on PubMed and ResearchGate.

**Results:**

34-year-old female patient presented in psychiatric outpatient clinic with low mood, lack of appetite, disgust against food, vomiting, dizziness and syncopal episodes. She noted anxiety, fear of death, fatigue and decrease in activity. She was apathetic, lacked motivation and had sleep disturbance. PHQ-9 - 18 points. Patient had hand tremor and ataxic gait. Weight 78kg, height 1.79m, BMI 24.34kg/m2.

Patient was diagnosed with morbid obesity in teenage years (130kg, 1.79m, BMI 40.57kg/m2). At the age of 23, gastric bypass surgery was done. After the operation patient was satisfied, lost weight. A few years later she felt depressed, apathetic and dropped out of university. Patient was reluctant to visit her GP. In 2021 patient’s vision worsened, gait became ataxic, appeared disgust against food, dizziness, several syncopal episodes. Patient was hospitalized in Neurological clinic due to suspected demyelinating central nervous system (CNS) disease. Patient was diagnosed with alimentary B12 and B9 deficiency, gastroesophageal reflux. She received treatment with Vitamins B12, B9. Demyelinating CNS disease was not confirmed. Patient became more depressed and anxious. She was diagnosed with depression and received treatment with escitalopram, later switched to venlafaxine, mirtazapine and phenibut. Little temporary improvement was observed, but patient had side-effects and still had vomiting and syncopal episodes.

At the time of Psychiatric outpatient visit, additional blood tests were done, revealing severe Vitamin D3 deficiency (3.96ng/ml). During treatment with fluvoxamine (50mg per day) in combination with olanzapine (5mg per day), vitamin B12, B9, D3 supplementation, patient‘s mood gradually improved, disgust against food disappeared, appetite improved, patient became more active, syncopal episodes disappeared and sleep improved. PHQ-9 after two months was 5 points.

**Conclusions:**

Patient with RDD and Vitamin B12, folate and D3 deficiency, disgust against food, vomiting, fainting, benefited from combination of fluvoxamine, olanzapine and vitamin supplementation. Dynamic monitoring of patients after gastric bypass surgeries and education on this topic is vital to ensure patient health. Further research is necessary on treatment combination strategies for RDD in case of vitamin deficiencies.

**Disclosure of Interest:**

None Declared

